# Haploidentical transplantation with post-transplant cyclophosphamide is not inferior to 9/10-MUD transplantation with ATG in patients with myeloid malignancies

**DOI:** 10.1038/s41409-026-02827-y

**Published:** 2026-04-08

**Authors:** Andrea Gantner, Svenja Labuhn, Daniel Fürst, Sophie Mannes, Sarah Flossdorf, Francis Ayuketang Ayuk, Thomas Schroeder, Matthias Stelljes, Robert Zeiser, Peter Dreger, Matthias Eder, Igor Wolfgang Blau, Johannes Schetelig, Arne Brecht, Andreas Burchert, Matthias Edinger, Verena Wais, Hubert Schrezenmeier, Hartmut Döhner, Sandra Schmeller, Katharina Fleischhauer, Nicolaus Kröger, Elisa Sala, German Registry for Hematopoietic Stem Cell Transplantation and cell Therapy, DRST

**Affiliations:** 1 Department of Internal Medicine III, University Hospital of Ulm, Ulm, Germany; 2 Institute of Statistics, University of Ulm, Ulm, Germany; 3 Department of Transplantation Immunology, Institute of Clinical Transfusion Medicine and Immunogenetics Ulm, Ulm, Germany; 4 German Red Cross Blood Transfusion Service, Baden Wuerttemberg - Hessen, University Hospital Ulm, Ulm, Germany; 5 German Registry for Hematopoietic Stem Cell Transplantation and Cell Therapy (DRST), Frankfurt, Germany; 6 Institute for Medical Informatics, Biometry and Epidemiology (IMIBE), University Hospital, University Duisburg-Essen, Essen, Germany; 7 Department of Stem Cell Transplantation, University Medical Center Hamburg-Eppendorf, Hamburg, Germany; 8 Department of Hematology and Stem Cell Transplantation, West German Cancer Center Essen, University Hospital Essen, Essen, Germany; 9 Department of Medicine A/Hematology and Oncology, University Hospital Münster, Münster, Germany; 10 Department of Medicine I/Hematology and Oncology, Faculty of Medicine, Freiburg University Medical Center, Freiburg, Germany; 11 Department of Hematology and Oncology, University Hospital Heidelberg, Heidelberg, Germany; 12 Department of Hematology, Hemostasis, Oncology, and Stem Cell Transplantation, Hannover Medical School, Hannover, Germany; 13 Medical Clinic, Charité University Medicine Berlin, Berlin, Germany; 14 Medizinische Klinik und Poliklinik I, Universitätsklinikum Carl Gustav Carus, Dresden, Germany; 15 DKD HELIOS Hospital Wiesbaden and HELIOS Dr. Horst Schmidt Hospitals Wiesbaden, Wiesbaden, Germany; 16 Department of Hematology, Oncology, and Immunology, Carreras Leukemia Center, Philips University Marburg and University Hospital Gießen and Marburg, Marburg, Germany; 17 Department of Internal Medicine III, University Clinic Regensburg, Regensburg, Germany; 18 Institute for Experimental Cellular Therapy, Essen University Hospital, Essen, Germany

**Keywords:** Bone marrow transplantation, Acute myeloid leukaemia

## Abstract

The selection of the best available donor is crucial for patients’ outcome after allogeneic stem cell transplantation (allo-SCT). In the absence of fully Human leucocyte antigen (HLA) -matched donors, mismatched unrelated donor (9/10-MUD) or haploidentical donor (haplo) can be considered. No consensus has been reached on the best alternative and large real-world data are warranted to support decisional processes. We compared the outcome of 1413 patients with myeloid malignancies undergoing allo-SCT from 9/10-MUD with anti-thymocyte-globulin (ATG) (*n* = 1134) or haplo with post-transplant cyclophosphamide (PT-Cy, *n* = 279) between 2009 and 2020 in 48 German centres. Donor type with related graft versus host disease (GvHD) prophylaxis showed in multivariable analysis no significant impact on acute GvHD development, both grade II-IV (HR 0.90, 95% CI 0.69–1.19, *p* = 0.469) and severe (HR 1.22, 95% CI 0.82-1.81, *p* = 0.319), nor on moderate to severe chronic GvHD (HR 0.78, 95% CI 0.59–1.03, *p* = 0.077). Moreover, no influence from donor type was observed on GVHD-relapse-free survival (HR 1.12, 95% CI 0.92–1.36, *p* = 0.227), progression-free survival (HR 1.2, 95% CI 0.95–1.51, *p* = 0.121), non-relapse mortality (HR 1.1, 95% CI 0.81–1.51, *p* = 0.542) and overall survival (HR 1.16, 95% CI 0.91–1.48, *p* = 0.235). Our real-world data demonstrate that haplo allo-SCT with PT-Cy is not inferior to 9/10-MUD allo-SCT with ATG.

## Introduction

Allogenic stem cell transplantation (allo-SCT) is the only potentially curative option for patients with high-risk haematological diseases. The number of transplanted patients is continuously growing due to constant improvement in toxicity profile [1–4]. Still, the outcome of allo-SCT remains influenced by non-relapse mortality (NRM), accounting for at least 20% of overall mortality within 3 years after transplantation [2]. One of the most common risk factors for NRM is the development of graft versus host disease (GvHD). The incidence of acute GvHD (aGvHD) and chronic GvHD (cGvHD) is 40–60% and 35–70%, respectively [5–7]. Despite the development of new drugs for its treatment in the last years [8–11], effective GvHD prevention remains crucial for the success of transplantation. In this context the choice of the best available donor plays an important role, as it has been shown to influence GVHD-relapse-free survival (GRFS), NRM, relapse incidence and overall survival (OS) [12, 13]. Human leucocyte antigen (HLA) identical related donors and matched unrelated donors (MUD) remain the preferred options but are only available in 16–75% of the cases [14]. In the absence of a fully HLA-matched donor, 9/10-matched unrelated donors (9/10-MUD) or HLA-haploidentical donors (haplo) can be considered [15–17]. The administration of post-transplant cyclophosphamide (PT-Cy) improved the outcome after haplo allo-SCT [18], contributing to a rapid increase in the number of haplo allo-SCT worldwide [16, 19]. Furthermore, several studies suggest that PT-Cy could be effective also in other settings, such as allo-SCT from 9/10-MUD [20–23] as well as from matched donors, both related [24, 25] and unrelated [26, 27]. More recently, large retrospective analyses have shown that PT-Cy in the 9/10-MUD setting can achieve outcomes comparable to those observed after MUD transplantation [28]. Nevertheless, anti-thymocyte-globulin (ATG)-based GVHD prophylaxis continues to represent a widely used standard approach in MUD (both 10/10 and 9/10) allo-SCT, as supported by current EBMT consensus recommendations [29]. In the absence of prospective randomised trials clearly establishing the superiority of PT-Cy over ATG in the 9/10-MUD setting, both strategies are currently employed in routine clinical practice. In this evolving therapeutic landscape, real-world data remain essential to inform the optimal choice of donor type and GVHD prophylaxis. We therefore conducted a multicentre retrospective study to compare the outcome of patients with myeloid malignancies undergoing 9/10-MUD allo-SCT using ATG versus haplo allo-SCT with PT-Cy on behalf of the German registry for hematopoietic stem cell transplantation and cell therapy (DRST). We performed sub-analyses to compare the outcomes of haplo + PT-Cy with 9/10-MUD + ATG across different sub-population stratifying according to the type of HLA mismatch. First, we analysed the sub-population with HLA class I mismatches, as mismatches at HLA-A, -B and -C loci proved to be associated with significantly worse OS compared to HLA class II mismatches [30]. A sub-analysis investigating haplo + PT-Cy versus 9/10-MUD + ATG allo-SCT with mismatch on the HLA-DQ locus was also performed. HLA-DQ mismatches are generally considered to have a smaller impact on overall mortality than other HLA mismatches [31–33], even though a meta-analysis reported increased mortality [34, 35].

## Patients and methods

### Data collection

We performed a retrospective study on consecutive patients undergoing allo-SCT for myeloid malignancies between 2009 and 2020, including data from 48 different German transplant centres. The study was approved by the ethics committee of the University of Ulm (Application No: 227/21). All participants gave written informed consent for data analysis. Inclusion criteria were: (1) confirmed diagnosis of myeloid malignancy with indication for allo-SCT; (2) adult age ( ≥ 18 years); (3) first allo-SCT from 9/10-MUD or haploidentical donor; (4) patients undergoing allo-SCT from 9/10-MUD must have received GvHD prophylaxis based on ATG, while patients transplanted from haploidentical donor must have received PT-Cy.

### Definitions

A 9/10-MUD was defined as a donor presenting a single HLA mismatch at one of five loci (HLA-A, -B, -C, -DRB1, -DQB1). A haplo donor was defined as family donor who shared one HLA haplotype with the recipient. The intensity of conditioning was defined as previously described [36, 37]. Neutrophil engraftment was defined as the first of three consecutive days with a neutrophil count ≥ 0.5 ×10^9/L, whereas platelet engraftment was defined as the first of three consecutive days with a platelet count ≥20 × 10⁹/L without platelet transfusion support for at least 7 days. Diagnosis and grading of aGVHD followed the 1994 Consensus Conference on Acute GVHD Grading [38], while diagnosis and grading of cGVHD were performed according to NIH Consensus Criteria [39, 40]. Hematopoietic stem cell transplantation comorbidity index (HCT-CI) and European Bone Marrow Transplantation (EBMT)-risk score were calculated as previously described [41, 42]. Disease stage was defined according to the EBMT-risk score [42]. OS was defined as the time from allo-SCT until death from any cause. Progression free Survival (PFS) was defined as time from allo-SCT to relapse/progress or death without prior relapse; surviving patients were censored at last follow-up. Non-relapse mortality (NRM) was defined as death not preceded by recurrent or progressive primary malignancy. GvHD-free-relapse-free survival (GRFS) was considered as the time from allo-SCT to one of the following events: aGVHD grade III-IV, cGvHD, relapse or death, whichever occurred first. The cumulative incidence of aGVHD or cGvHD were defined as the number of days from allo-SCT to first occurrence of aGVHD by day +100 or cGvHD by 1 year after transplant, with death from any other cause as competing event.

### Statistical analysis

Wilcoxon-Mann-Whitney U-Test was used for continuous variables and chi-square testing for categorical ones. Survival rates for univariable analysis were calculated using the Kaplan-Meier method with log-rank significance testing. Cumulative incidences were evaluated for competing endpoints with competing risk statistics according to the Fine Gray model. For the multivariable analysis (MVA) Cox’s proportional hazards model was used to assess the effect of possible prognostic factors on OS, PFS, NRM and GRFS. Following covariables were considered: donor type (9/10-MUD versus haplo), patient age, donor age, disease status at transplant according to the EBMT risk-score stratification (early, intermediate, advanced) [42], Karnofsky performance status (KPS), year of allo-SCT, graft source, sex mismatch, conditioning intensity, total body irradiation (TBI), HCT-CI and cytomegalovirus (CMV) status. Missing values were treated as separate categories. As additional validation, a 1:1 propensity score matched-pair analysis was performed for the same endpoints (Supplementary Methods). Statistical analysis was performed with the Software R version 4.4.1.

## Results

### Patients’ and transplants’ characteristics

A total of 1413 patients undergoing allo-SCT fulfilled the inclusion criteria. Two hundred seventy-nine (19.8%) were transplanted from haplo donor with PT-Cy, while 1134 (80.2%) received 9/10-MUD with ATG. Patients’ and transplants’ characteristics are listed in Table 1.

**Table 1:** Patients´ and transplantations´ characteristics.

Variable	Values	Total	9/10-MUD + ATG	Haplo + PT-Cy	*P* value
Number	Number, n (%)	1413	1134 (80.2%)	279 (19.8%)	
Patient age at allo-SCT	Years, median (range)	58 (18-79)	58 (18-77)	58 (18-79)	0.995
Disease Type	AML, n (%)	1099 (77.8%)	866 (76.4%)	233 (83.5%)	**0.013**
	MDS, n (%)	314 (22.2%)	268 (23.6%)	46 (16.5%)	
Disease status at allo-SCT*	Early, n (%)	677 (47.9%)	535 (47.2%)	142 (50.9%)	0.128
	Intermediate, n (%)	371 (26.3%)	304 (26.8%)	67 (24%)	
	Advanced, n (%)	362 (25.6%)	294 (25.9%)	68 (24.4%)	
	Missing, n (%)	3 (0.2%)	1 (0.1%)	2 (0.7%)	
HCT-CI	0-1, n (%)	533 (37.7%)	384 (33.9%)	149 (53.4%)	**<0.001**
	2-3, n (%)	246 (17.4%)	167 (14.7%)	79 (28.3%)	
	≥ 4, n (%)	142 (10.1%)	98 (8.6%)	44 (15.8%)	
	Missing, n (%)	492 (34.8%)	485 (42.8%)	7 (2.5%)	
Donor age	≤35 years, n (%)	427 (30.2%)	302 (26.6%)	125 (44.8%)	**<0.001**
	>35 years, n (%)	377 (26.7%)	238 (21%)	139 (49.8%)	
	Missing, n (%)	609 (43.1%)	594 (52.4%)	15 (5.4%)	
Sex mismatch patient/donor	Yes, n (%)	204 (14.4%)	144 (12.7%)	60 (21.5%)	**<0.001**
	No, n (%)	1150 (81.4%)	931 (82.1%)	219 (78.5%)	
	Missing, n (%)	59 (4.2%)	59 (5.2%)	0 (0%)	
CMV status patient/donor	Neg/neg, n (%)	379 (26.8%)	309 (27.2%)	70 (25.1%)	**<0.001**
	Pos/pos, n (%)	528 (37.5%)	395 (34.8%)	133 (47.7%)	
	Pos/neg, n (%)	330 (23.3%)	274 (24.2%)	56 (20.1%)	
	Neg/pos, n (%)	154 (10.9%)	135 (11.9%)	19 (6.8%)	
	Missing, n (%)	22 (1.5%)	21 (1.9%)	1 (0.3%)	
Conditioning regimen	MAC, n (%)	737 (52.2%)	580 (51.2%)	157 (56.3%)	0.165
	RIC, n (%)	670 (47.4%)	548 (48.3%)	122 (43.7%)	
	Missing, n (%)	6 (0.4%)	6 (0.5%)	0 (0%)	
TBI	Yes, n (%)	326 (23.1%)	282 (24.9%)	44 (15.8%)	**0.004**
	No, n (%)	1078 (76.3%)	846 (74.6%)	232 (83.2%)	
	Missing, n (%)	9 (0.6%)	6 (0.5%)	3 (1.1%)	
Stem cell source	BM, n (%)	126 (8.9%)	55 (4.8%)	71 (25.4%)	**<0.001**
	PBSC, n (%)	1283 (90.8%)	1078 (95.1%)	205 (73.5%)	
	Missing, n (%)	4 (0.3%)	1 (0.1%)	3 (1.1%)	
Additional GvHD-prophylaxis	CNI + MMF, n (%)	810 (57.3%)	558 (49.2%)	252 (90.3%)	**<0.001**
	CNI + MTX, n (%)	436 (30.9%)	436 (38.4%)	0 (0%)	
	CNI, n (%)	117 (8.3%)	107 (9.5%)	10 (3.6%)	
	Other, n (%)	33 (2.3%)	16 (1.4%)	17 (6.1%)	
	Missing, n (%)	17 (1.2%)	17 (1.5%)	0 (0%)	
Year of allo-SCT	2000-2005, n (%)	17 (1.2%)	17 (1.5%)	0 (0%)	**<0.001**
	2006-2010, n (%)	347 (24.6%)	346 (30.5%)	1 (0.4%)	
	2011-2015, n (%)	484 (34.2%)	449 (39.6%)	35 (12.5%)	
	2016-2020, n (%)	565 (40%)	322 (28.4%)	243 (87.1%)	

*allo-SCT* allogeneic stem cell transplantation, *MUD* matched unrelated donor, *ATG* anti-thymocyte globulin, *Haplo* haploidentical donor, *PT-CY* Post transplant cyclophosphamide, *AML* acute myeloid leukemia, *MDS* myelodysplastic syndrome, *HCT-CI* hematopoietic cell transplantation comorbidity index, *MAC* Myeloablative conditioning, *RIC* Reduced intensity conditioning, *TBI* Total body irradiation, *BM* Bone Marrow, *PBSC* Peripheral blood stem cells, *CNI* calcineurin inhibitor, *MMF* mycophenolate mophetil, *MTX* methotrexate.

^*^According to the classification present in the EBMT-risk score.

Patients’ age, disease status by EBMT-risk score and intensity of conditioning were homogeneously represented between the two groups (9/10-MUD versus haplo). Patients undergoing haplo allo-SCT were transplanted more recently (87.1% between 2016 and 2020), whereas the distribution of 9/10-MUD allo-SCT was quite homogeneous over time. Moreover, bone marrow was more commonly used as stem cell source in the haplo cohort (25.4% versus 4.8%, respectively – *p* < 0.001).

### Outcome analysis

**Fig. 1:**
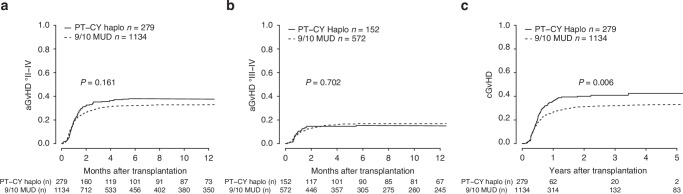
Cumulative incidence of acute and chronic GvHD in the 9/10-MUD + ATG cohort compared with the haplo + PT-Cy cohort. **Panel 1 a**. Cumulative incidence of aGVHD grade II to IV (requiring treatment) at day +100 post allo-SCT: 30.5% in the 9/10-MUD cohort versus 35.6% in the haplo-cohort, *p* = 0,161. **Panel 1b**. Cumulative incidence of severe aGVHD (grade III-IV) at day +100: 15.4% in the 9/10-MUD cohort versus 14.8% in the haplo cohort, *p* = 0,702. **Panel 1 c**. Cumulative incidence of cGvHD at 1 year after allo-SCT was significantly higher in the haplo group (36.1%) as compared to the 9/10-MUD cohort (26.8%), *p* = 0.006.

**Fig. 2:**
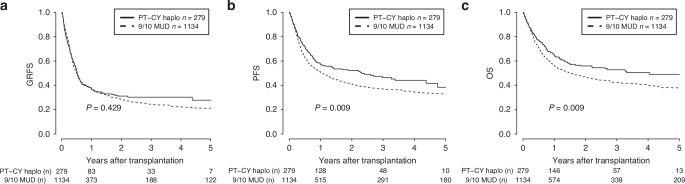
Univariable analysis for overall survival (OS), progression free survival (PFS) and GvHD-free-relapse-free survival (GRFS) comparing the haplo + PT-Cy cohort with the 9/10-MUD + ATG cohort. **Panel 2 a**. 5y-GRFS: 22.5% in the 9/10-MUD cohort versus 24.7% in the haplo cohort, *p* = 0.774. **Panel 2b**. 5y-PFS: 38.4% in the haplo cohort versus 32.9% in the 9/10-MUD cohort, *p* = 0.009. **Panel 2 c**. 5y-OS: 49% in the haplo cohort versus 37.8% in the 9/10-MUD cohort, *p* = 0.009.

The cumulative incidence of neutrophil engraftment at day +28 after allo-SCT was significantly higher in the 9/10-MUD cohort (90% versus 85%, *p* < 0.001). Similarly, the cumulative incidence of platelet engraftment by day +28 was higher in the 9/10-MUD cohort (84.4% versus 61.2%, *p* < 0.001). The cumulative incidence of grade II-IV aGVHD at day +100 post allo-SCT was 30.5% in the 9/10-MUD cohort versus 35.6% in the haplo cohort (*p* = 0.161). Similarly, the day +100 cumulative incidence of severe aGVHD (grade III-IV) did not differ significantly between the two groups (15.4% in the 9/10-MUD cohort versus 14.8% in the haplo cohort, *p* = 0.702). Conversely, one year after allo-SCT the risk of cGVHD was significantly higher in the haplo group (36.1%) compared to the 9/10-MUD cohort (26.8%, *p* = 0.006). Figure 1. The cumulative incidence of disease relapse five years after allo-SCT was comparable between the two sub-populations (30.3% in the 9/10-MUD cohort versus 32.2% in the haplo cohort, *p* = 0.378). In the univariable analysis, the 5y-GRFS was similar, 21.1% in the 9/10-MUD cohort versus 27.8% in the haplo one (*p* = 0.429). The 5y-PFS was significantly better in the haplo-group (38.4%) as compared to the 9/10-MUD one (32.9%), *p* = 0.009. This resulted in a significantly better 5y-OS for patients receiving haplo allo-SCT (49.0%) compared to the 9/10-MUD option (37.8%), *p* = 0.009 Fig. 2.

In the MVA, considering the occurrence of grade II-IV aGvHD, the type of donor with the associated GvHD prophylaxis (9/10-MUD plus ATG versus haplo plus PT-Cy) did not have a significant influence (HR 0.90, 95% confidence interval (CI) 0.69–1.19, *p* = 0.469). The same applied for grade III-IV aGvHD (HR 1.22, 95% CI 0.82–1.81, *p* = 0.319). Considering the development of treatment-requiring cGvHD (moderate to severe), the donor type showed again no significant impact (HR 0.78, 95% CI 0.59–1.03, *p* = 0.077). When evaluating GRFS as endpoint, the donor type with associated GvHD prophylaxis (9/10-MUD plus ATG versus haplo plus PT-Cy) continued to show no significant influence (HR 1.12, 95% CI 0.92–1.36, *p* = 0.227). However, advanced disease, donor/patient sex mismatch and patient CMV seropositivity emerged as independent risk factors (Table 2). The same was observed for NRM (9/10-MUD plus ATG versus haplo plus PT-Cy: HR 1.1, 95% CI 0.81–1.51, *p* = 0.542), which was negatively influenced by advanced disease, older patient age, patient CMV seropositivity, poor KPS and the use of TBI. Among NRM-related deaths, infection was the most common cause, with no significant differences between the two groups (9/10-MUD + ATG: 53.2% versus haplo + PT-Cy: 45.6%; *p* = 0.29). Similar results were observed for GvHD-related NRM (29.0% versus 37.1%, respectively, *p* = 0.29) and other causes (17.7% versus 17.4%, respectively, p = 0.95). For PFS, there was still no role played by the type of donor and related GvHD prophylaxis (HR 1.2, 95% CI 0.95–1.51, *p* = 0.121). A negative impact was exerted by patient advanced age and disease, as well as poor KPS and patient CMV seropositivity (Table 2). Finally, also in case of OS we could confirm the absence of an impact from donor type with combined GvHD prophylaxis (9/10-MUD plus ATG versus haplo plus PT-Cy: HR 1.16, 95% CI 0.91–1.48, *p* = 0.235). Conversely, patient age, advanced disease, poor KPS, donor/patient sex mismatch, patient CMV seropositivity, as well as the use of TBI demonstrated a negative impact on patients’ survival after allo-SCT (Table 2). The matched-pair analysis confirmed these results, in particular the absence of influence from the donor type with associated GvHD prophylaxis on survival outcomes (Supplementary Materials – Table S1).

**Table 2:** Multivariable analysis of factors influencing overall survival (OS), progression free survival (PFS) and GvHD-free-relapse-free survival (GRFS) after allogenic stem cell transplantation (allo-SCT).

	OS			PFS			GRFS		
	HR	95% CI	*P* value	HR	95% CI	*P* value	HR	95% CI	*P* value
Donor type									
**9/10-MUD** + **ATG**	-	-		-	-		-	-	
**Haplo + PT-Cy**	1.16	0.91-1.48	0.235	1.2	0.95-1.51	0.121	1.12	0.92-1.36	0.227
Patient age at allo-SCT	1.02	1.01-1.03	**<0.001**	1.01	1.01-1.02	**<0.001**	1.01	1.0-1.02	**0.001**
Disease status at allo-SCT*									
**early**	-	-		-	-		-	-	
**Intermediate**	1.16	0.97-1.39	0.092	1.18	1.0-1.4	**0.05**	1.04	0.89-1.22	0.608
**advanced**	1.7	1.43-2.03	**<0.001**	1.69	1.43-2	**<0.001**	1.6	1.37-1.86	**<0.001**
KPS < 80	1.49	1.15-1.92	**0.003**	1.41	1.1-1.82	**0.007**	1.2	0.94-1.53	0.138
Stem cell source									
**PBSC**	-	-		-	-		-	-	
**BM**	0.91	0.7-1.2	0.511	0.97	0.75-1.26	0.818	0.91	0.72-1.15	0.427
Sex mismatch (donor/patient)									
**others**	-	-		-	-		-	-	
**Female to male**	1.28	1.05-1.56	**0.015**	1.2	0.99-1.46	0.061	1.25	1.05-1.5	**0.012**
Donor age									
**>35 years**	1.2	0.99-1.46	0.07	1.2	0.99-1.45	0.057	1.06	0.9-1.25	0.462
Conditioning regimen									
**MAC**	-	-		-	-		-	-	
**RIC**	0.94	0.81-1.1	0.457	0.97	0.83-1.12	0.637	0.97	0.85-1.11	0.648
CMV status (patient/donor)									
**neg/neg**	-	-		-	-		-	-	
**neg/pos**	1.12	0.86-1.46	0.388	1.06	0.82-1.37	0.635	1.1	0.88-1.38	0.399
**pos/neg**	1.39	1.14-1.69	**0.001**	1.38	1.14-1.67	**0.001**	1.24	1.04-1.48	**0.018**
**pos/pos**	1.27	1.06-1.53	**0.011**	1.25	1.06-1.49	**0.013**	1.17	0.99-1.37	0.06
TBI									
**no**	-	-		-	-		-	-	
**yes**	1.2	1.01-1.44	**0.039**	1.05	0.89-1.25	0.56	0.95	0.81-1.12	0.545
Year of allo-SCT	0.99	0.97-1.01	0.328	0.99	0.97-1.01	0.249	0.99	0.97-1.01	0319

*MUD* matched unrelated donor, *ATG* anti-thymocyte globulin, *Haplo* haploidentical donor, *PT-CY* Post transplant cyclophosphamide, *allo-SCT* allogeneic stem cell transplantation, *KPS* Karnofsky Performance Status, *PBSC* Peripheral blood stem cells, *BM* Bone Marrow, *MAC* Myeloablative conditioning, *RIC* Reduced intensity conditioning, *TBI* Total body irradiation.

^*^According to the classification present in the EBMT-risk score.

Furthermore, even when considering the impact of HLA class I mismatches in the setting of 9/10-MUD + ATG allo-SCT compared to haplo + PT-Cy, the type of donor with combined GvHD prophylaxis did not affect the occurrence of grade II-IV and grade III-IV aGvHD (HR 1.01, 95% CI 0.76–1.34, *p* = 0.939 and HR 1.39, 95% CI 0.92–2.08, *p* = 0.116, respectively), moderate to severe cGvHD (HR 0.78, 95% CI 0.58–1.05, *p* = 0.099), as well as allo-SCT major outcomes (OS, PFS, GRFS – Table S2).

### Sub-analysis: the DQ mismatch cohort

In the sub-analysis comparing 9/10-MUD with a DQ mismatch and ATG (*n* = 136) with the haplo plus PT-Cy cohort (*n* = 279), the cumulative incidence of aGvHD requiring treatment (grade II-IV) by day +100 after allo-SCT was significantly lower in the 9/10-MUD with DQ mismatch group (24.6% versus 38.0% in the haplo group, *p* = 0.008), whereas the day +100 cumulative incidence of grade III-IV aGVHD was not statistically different among the two sub-population (12.8% for the 9/10-MUD with DQ mismatch group versus 16.0% for the haplo group, *p* = 0.366). Similarly, the 1-year cumulative incidence of cGvHD was not statistically different, with 26.2% in the 9/10-MUD with DQ mismatch group versus 36.1% in the haplo cohort (*p* = 0.091). In the MVA for GRFS the only factor demonstrating an impact on outcome was advanced disease at the time of allo-SCT (HR 1.62, 95% CI 1.19–2.2, *p* = 0.002). All other variables, including the type of donor with associated GvHD prophylaxis (9/10-MUD with DQ mismatch plus ATG versus haplo plus PT-Cy) did not appear to significantly influence this endpoint (Table 3). Moreover, donor type did not have an impact on PFS and OS (HR 1.22, 95% CI 0.79–1.9, *p* = 0.384 and HR 1.05, 95% CI 0.66-1.66, p = 0.85 respectively). In both cases, patient older age, advanced disease, poor KPS, and CMV seropositivity were associated with worse outcomes (Table 3).

**Table 3:** Multivariable analysis of factors influencing overall survival (OS).

	OS			PFS			GRFS		
	HR	95% CI	*P* value	HR	95% CI	*P* value	HR	95% CI	*P* value
Donor type									
**9/10-MUD** + **ATG**	-	-		-	-		-	-	
**Haplo + PT-Cy**	1.05	0.66-1.66	0.85	1.22	0.79-1.9	0.384	1.01	0.68-1.5	0.961
Patient age at allo-SCT	1.02	1.01-1.04	**0.001**	1.02	1-1.03	**0.014**	1	0.99-1.01	0.645
Disease status at allo-SCT*									
**early**	-	-		-	-		-	-	
**Intermediate**	1.1	0.74-1.62	0.637	1.21	0.84-1.73	0.308	1.14	0.83-1.56	0.436
**advanced**	2.02	1.41-2.86	**<0.001**	2.07	1.48-2.89	**<0.001**	1.62	1.19-2.2	**0.002**
KPS < 80	2.15	1.33-3.48	**0.002**	1.96	1.23-3.13	**0.005**	1.39	0.89-2.15	0.144
Stem cell source									
**PBSC**	-	-		-	-		-	-	
**BM**	0.88	0.59-1.33	0.545	0.95	0.65-1.4	0.813	1.12	0.82-1.55	0.472
Sex mismatch (donor/patient)									
**others**	-	-		-	-		-	-	
**Female to male**	1.45	1.01-2.09	**0.046**	1.25	0.88-1.78	0.208	1.16	0.84-1.59	0.366
Donor age									
**>35 years**	1.35	0.94-1.93	0.108	1.48	1.05-2.07	**0.024**	1.24	0.92-1.65	0.153
Conditioning regimen									
**MAC**	-	-		-	-		-	-	
**RIC**	1.22	0.89-1.69	0.221	1.13	0.83-1.53	0.429	1.26	0.96-1.65	0.101
CMV status (patient/donor)									
**neg /neg**	-	-		-	-		-	-	
**neg/pos**	1.69	0.94-3.05	0.081	1.71	0.96-3.05	0.066	1.12	0.68-1.83	0.664
**pos/neg**	1.33	0.86-2.04	0.196	1.56	1.04-2.36	**0.033**	1.09	0.75-1.58	0.657
**pos/pos**	1.55	1.05-2.27	**0.026**	1.62	1.12-2.33	**0.01**	1.25	0.91-1.72	0.162
TBI									
**none**	-	-		-	-		-	-	
**Yes**	1.32	0.91-1.93	0.143	1.18	0.82-1.69	0.368	1.07	0.78-1.49	0.666
Year of allo-SCT	1.05	0.98-1.12	0.147	1.04	0.98-1.1	0.158	1.04	0.98-1.09	0.153

*MUD* matched unrelated donor, *ATG* anti-thymocyte globulin, *Haplo* haploidentical donor, *PT-CY* Post transplant cyclophosphamide, *allo-SCT* allogeneic stem cell transplantation, *KPS* Karnofsky Performance Status, *PBSC* Peripheral blood stem cells, *BM* Bone Marrow, *MAC* Myeloablative conditioning, *RIC* Reduced intensity conditioning, *TBI* Total body irradiation.

^*^According to the classification present in the EBMT-risk score.

Progression free survival (PFS) and GvHD-free-relapse-free survival (GRFS) after allogenic stem cell transplantation (allo-SCT). DQ mismatch cohort.

## Discussion

In this large, multicentre, registry-based study, we retrospectively analysed the outcome of 1413 patients undergoing allo-SCT from a 9/10-MUD or a haplo donor for myeloid malignancies, over a 10-years period across 48 transplant centres in Germany. The aim of our analysis was to compare the outcome 9/10-MUD with ATG-based GvHD prophylaxis allo-SCT to that of haplo transplantation with PT-Cy to help address the question which donor type and GvHD prophylaxis should be preferred in the absence of a fully HLA-matched donor. This is a clinically relevant question, given that the administration of PT-Cy improved the outcome of haplo allo-SCT over the years [18]. Several studies demonstrated that haplo allo-SCT is associated with an acceptable safety profile and a clinical outcome comparable to what observed with HLA-matched allo-SCT [43–45]. Moreover, it has been shown, also in prospective randomised clinical trials [26], that PT-Cy could be an effective and safe strategy also in other donor contexts [20, 21, 23–26, 28]. However, ATG in association with CNI and methotrexate remains one of the most widely used regimens for GvHD prophylaxis in unrelated donor allo-SCT, both matched and mismatched [29]. Additionally, a recent published work [46] demonstrated inferior outcome in terms of survival for patients undergoing 9/10-MUD allo-SCT compared to fully HLA-matched donor transplantation, especially in case of HLA class I mismatches and without significant differences between non-PT-Cy and PT-Cy approach. This suggests that HLA matching remains clinically relevant in the era of PT-Cy, and that GvHD prophylaxis platforms continue to play a crucial role in modulating transplant-related toxicities.

In light of this evolving landscape, our analysis showed that (1) the cumulative incidence of aGvHD at day +100 after allo-SCT was not statistically different in the two subgroups (9/10-MUD plus ATG versus haplo plus PT-Cy); (2) the cumulative incidence of cGvHD at 1 year after allo-SCT was higher in the haplo + PT-Cy cohort compared to the 9/10-MUD + ATG group (36.1% versus 26.8%, *p* = 0.006), but this was not confirmed by the MVA, especially when analysing cGvHD requiring treatment (moderate and severe according to the NIH criteria [39, 40] – HR 0.78, 95% CI 0.59-1.03, *p* = 0.077); (3) in the MVA, we did not observe any statistically significant impact of the donor type combined with the associated GvHD prophylaxis platform (9/10-MUD + ATG versus haplo + PT-Cy) on any of the major outcome endpoints, including GRFS, NRM, PFS and OS, as well as on the occurrence of treatment-requiring a- and cGvHD; (4) these results were confirmed also considering the sub-population of patients undergoing allo-SCT from a 9/10-MUD with a mismatch on HLA class I loci as well as on DQ locus compared to haplo cohort.

Our results confirm on a large and coherent basis data emerging from previous studies. Baker [47] compared the outcome of haplo allo-SCT with PT-Cy (*n* = 54) with transplantation from unrelated donor plus ATG (*n* = 59). No differences were observed regarding incidence of a- and cGvHD, PFS and OS. In a similar analysis [48] with a cohort of 781 patients with ALL in complete remission at allo-SCT, where patients undergoing haplo allo-SCT received mostly PT-Cy (only 8% received ATG), and 21% of patients in the 9/10-MUD cohort had ATG, no significant differences were observed considering GRFS and OS. Finally, Baron et al. [49] compared the outcome of 9/10-MUD versus haplo in AML with active disease at allo-SCT and found similar NRM, leukemia free survival, and OS 2 years after transplantation. These data confirm comparable outcome for haplo- and 9/10-MUD allo-SCTs.

There are some limitations in our study. The first one is the retrospective nature of this registry-based analysis. Secondly, the two cohorts (9/10-MUD and haplo) were not fully balanced considering baseline characteristics. For instance, due to the more recent adoption of the haplo PT-Cy platform, most patients in the haplo cohort (87%) were transplanted between 2016 and 2020, whereas only 28% of the 9/10-MUD cohort were transplanted in the same time frame. Given the improvements in supportive treatments and conditioning strategies over the last decades, this imbalance may have influenced the outcome. However, we included the variable “year of transplantation” in the MVA and found no impact on the outcome. Furthermore, due to the retrospective, registry-based nature of our dataset, some important variables that could have strengthened our analysis—such as minimal residual disease status at the time of allo-SCT, detailed molecular and genetic information of the underlying disease, and data on immune reconstitution and infections —were not available. In addition, information on dose and formulation of ATG was not reported in the primary data with sufficient granularity to allow for statistical evaluation. Finally, due to the lack of availability in register of detailed HLA-typing data, we were unable to evaluate the impact of specific HLA mismatches on outcome according to previously published data, both for 9/10-MUD allo-SCT considering the central role of graft-versus-host peptide-binding motives mismatches [46, 50], and in the haplo setting [51].

Nevertheless, the present study is, to our knowledge, one of the largest multicenter comparisons between haplo allo-SCT with PT-Cy and 9/10-MUD allo-SCT with ATG-based GvHD prophylaxis. Taken together our data demonstrate, on a retrospective basis, that the outcome of haplo allo-SCT plus PT-Cy is not inferior to the outcome of 9/10-MUD plus ATG allo-SCT, in terms of toxicity, GvHD incidence, PFS and, ultimately, OS. This is a clinically relevant finding, especially considering that prospective trials aiming to compare haploidentical transplantation with PT-Cy versus 9/10 MUD with ATG have preliminarily confirmed our results but failed to complete accrual [52]. This further underscores the importance of our study as robust real-world evidence in the absence of completed randomised trials. As donor selection remains fundamental for allo-SCT, according to our data, if no fully HLA-matched donor is available, the choice of an haplo donor with PT-Cy can be a valid option over a 9/10-MUD with ATG. This approach may facilitate more timely transplantation while maintaining comparable efficacy and safety. Prospective randomised studies would be needed for further validation.

## Supplementary information

Supplemental Material

Appendix

## Data Availability

The dataset generated during and/or analysed during the current study are available from the corresponding author on reasonable request.

## References

[1] Horan JT, Logan BR, Agovi-Johnson MA, Lazarus HM, Bacigalupo AA, Ballen KK, et al. Reducing the risk for transplantation-related mortality after allogeneic hematopoietic cell transplantation: how much progress has been made? J Clin Oncol. 2011;29:805–13. 21220593 10.1200/JCO.2010.32.5001 PMC3068057

[2] Barrett J. Why is a 3-year NRM following allogeneic transplantation still stuck at approximately 20%? Best Pr Res Clin Haematol. 2018;31:414–9. 10.1016/j.beha.2018.09.011 30466759

[3] Savani BN, Labopin M, Kröger N, Finke J, Ehninger G, Niederwieser D, et al. Expanding transplant options to patients over 50 years. Improved outcome after reduced intensity conditioning mismatched-unrelated donor transplantation for patients with acute myeloid leukemia: a report from the Acute Leukemia Working Party of the EBMT. Haematologica. 2016;101:773–80. 26969081 10.3324/haematol.2015.138180 PMC5013965

[4] McDonald GB, Sandmaier BM, Mielcarek M, Sorror M, Pergam SA, Cheng GS, et al. Survival, Nonrelapse Mortality, and Relapse-Related Mortality After Allogeneic Hematopoietic Cell Transplantation: Comparing 2003-2007 Versus 2013-2017 Cohorts. Ann Intern Med. 2020;172:229–39. 31958813 10.7326/M19-2936 PMC7847247

[5] Jamy O, Zeiser R, Chen YB. Novel developments in the prophylaxis and treatment of acute GVHD. Blood. 2023;142:1037–46. 37471585 10.1182/blood.2023020073

[6] Zeiser R. Novel Approaches to the Treatment of Chronic Graft-Versus-Host Disease. J Clin Oncol. 2023;41:1820–4. 36800551 10.1200/JCO.22.02256

[7] Axt L, Naumann A, Toennies J, Haen SP, Vogel W, Schneidawind D, et al. Retrospective single center analysis of outcome, risk factors and therapy in steroid refractory graft-versus-host disease after allogeneic hematopoietic cell transplantation. Bone Marrow Transpl. 2019;54:1805–14. 10.1038/s41409-019-0544-y 31089279

[8] Zeiser R, Polverelli N, Ram R, Hashmi SK, Chakraverty R, Middeke JM, et al. Ruxolitinib for Glucocorticoid-Refractory Chronic Graft-versus-Host Disease. N Engl J Med. 2021;385:228–38. 34260836 10.1056/NEJMoa2033122

[9] Zeiser R, von Bubnoff N, Butler J, Mohty M, Niederwieser D, Or R, et al. Ruxolitinib for Glucocorticoid-Refractory Acute Graft-versus-Host Disease. N Engl J Med. 2020;382:1800–10. 32320566 10.1056/NEJMoa1917635

[10] Cutler C, Lee SJ, Arai S, Rotta M, Zoghi B, Lazaryan A, et al. Belumosudil for chronic graft-versus-host disease after 2 or more prior lines of therapy: the ROCKstar Study. Blood. 2021;138:2278–89. 34265047 10.1182/blood.2021012021 PMC8641099

[11] Wolff D, Cutler C, Lee SJ, Pusic I, Bittencourt H, White J, et al. Axatilimab in Recurrent or Refractory Chronic Graft-versus-Host Disease. N Engl J Med. 2024;391:1002–14. 39292927 10.1056/NEJMoa2401537

[12] Kollman C, Spellman SR, Zhang MJ, Hassebroek A, Anasetti C, Antin JH, et al. The effect of donor characteristics on survival after unrelated donor transplantation for hematologic malignancy. Blood. 2016;127:260–7. 26527675 10.1182/blood-2015-08-663823 PMC4713163

[13] Passweg JR, Zhang MJ, Rocha V, Kan F, Champlin RE, Isola LM, et al. Donor characteristics affecting graft failure, graft-versus-host disease, and survival after unrelated donor transplantation with reduced-intensity conditioning for hematologic malignancies. Biol Blood Marrow Transpl. 2011;17:1869–73. 10.1016/j.bbmt.2011.07.008 PMC3217167 21771571

[14] Gragert L, Eapen M, Williams E, Freeman J, Spellman S, Baitty R, et al. HLA match likelihoods for hematopoietic stem-cell grafts in the U.S. registry. N Engl J Med. 2014;371:339–48. 25054717 10.1056/NEJMsa1311707 PMC5965695

[15] Lee CJ, Savani BN, Mohty M, Labopin M, Ruggeri A, Schmid C, et al. Haploidentical hematopoietic cell transplantation for adult acute myeloid leukemia: a position statement from the Acute Leukemia Working Party of the European Society for Blood and Marrow Transplantation. Haematologica. 2017;102:1810–22. 28883081 10.3324/haematol.2017.176107 PMC5664385

[16] Passweg JR, Baldomero H, Bader P, Bonini C, Duarte RF, Dufour C, et al. Use of haploidentical stem cell transplantation continues to increase: the 2015 European Society for Blood and Marrow Transplant activity survey report. Bone Marrow Transpl. 2017;52:811–7. 10.1038/bmt.2017.34 PMC5467246 28287639

[17] Brissot E, Labopin M, Stelljes M, Ehninger G, Schwerdtfeger R, Finke J, et al. Comparison of matched sibling donors versus unrelated donors in allogeneic stem cell transplantation for primary refractory acute myeloid leukemia: a study on behalf of the Acute Leukemia Working Party of the EBMT. J Hematol Oncol. 2017;10:130. 28646908 10.1186/s13045-017-0498-8 PMC5483262

[18] Luznik L, O’Donnell PV, Symons HJ, Chen AR, Leffell MS, Zahurak M, et al. HLA-haploidentical bone marrow transplantation for hematologic malignancies using nonmyeloablative conditioning and high-dose, posttransplantation cyclophosphamide. Biol Blood Marrow Transpl. 2008;14:641–50. 10.1016/j.bbmt.2008.03.005 PMC2633246 18489989

[19] Passweg JR, Baldomero H, Alexander T, Angelucci E, Averbuch D, Bazarbachi A, et al. Utilization of hematopoietic cell transplantation and cellular therapy technology in Europe and associated Countries. Using the 2022 activity survey data to correlate with economic and demographic factors. A report from the EBMT. Bone Marrow Transpl. 2025;60:227–36. 10.1038/s41409-024-02459-0 PMC11810786 39578528

[20] Battipaglia G, Labopin M, Kröger N, Vitek A, Afanasyev B, Hilgendorf I, et al. Posttransplant cyclophosphamide vs antithymocyte globulin in HLA-mismatched unrelated donor transplantation. Blood. 2019;134:892–9. 31270102 10.1182/blood.2019000487

[21] Al Malki MM, Tsai NC, Palmer J, Mokhtari S, Tsai W, Cao T, et al. Posttransplant cyclophosphamide as GVHD prophylaxis for peripheral blood stem cell HLA-mismatched unrelated donor transplant. Blood Adv. 2021;5:2650–9. 34156440 10.1182/bloodadvances.2021004192 PMC8270662

[22] Shaw BE, Jimenez-Jimenez AM, Burns LJ, Logan BR, Khimani F, Shaffer BC, et al. National Marrow Donor Program-Sponsored Multicenter, Phase II Trial of HLA-Mismatched Unrelated Donor Bone Marrow Transplantation Using Post-Transplant Cyclophosphamide. J Clin Oncol. 2021;39:1971–82. 33905264 10.1200/JCO.20.03502 PMC8260905

[23] Kasamon YL, Ambinder RF, Fuchs EJ, Zahurak M, Rosner GL, Bolaños-Meade J, et al. Prospective study of nonmyeloablative, HLA-mismatched unrelated BMT with high-dose posttransplantation cyclophosphamide. Blood Adv. 2017;1:288–92. 29242852 10.1182/bloodadvances.2016002766 PMC5726600

[24] Desai N, Altareb M, Remberger M, Chen C, Alfaro Moya T, Al-Shaibani E, et al. PTCy-based graft-versus-host disease prophylaxis for matched sibling donor allogeneic hematopoietic cell transplantation. Blood Adv. 2025;9:660–9. 39565954 10.1182/bloodadvances.2024014781 PMC11881745

[25] Curtis DJ, Patil SS, Reynolds J, Purtill D, Lewis C, Ritchie DS, et al. Graft-versus-Host Disease Prophylaxis with Cyclophosphamide and Cyclosporin. N Engl J Med. 2025;393:243–54. 40513032 10.1056/NEJMoa2503189 PMC13186568

[26] Bolaños-Meade J, Hamadani M, Wu J, Al Malki MM, Martens MJ, Runaas L, et al. Post-Transplantation Cyclophosphamide-Based Graft-versus-Host Disease Prophylaxis. N Engl J Med. 2023;388:2338–48. 37342922 10.1056/NEJMoa2215943 PMC10575613

[27] Brissot E, Labopin M, Labussière H, Fossard G, Chevallier P, Guillaume T, et al. Post-transplant cyclophosphamide versus anti-thymocyte globulin after reduced intensity peripheral blood allogeneic cell transplantation in recipients of matched sibling or 10/10 HLA matched unrelated donors: final analysis of a randomized, open-label, multicenter, phase 2 trial. Blood Cancer J. 2024;14:31. 38374026 10.1038/s41408-024-00990-3 PMC10876658

[28] Shaffer BC, Gooptu M, DeFor TE, Maiers M, Bolaños-Meade J, Abboud R, et al. Post-Transplant Cyclophosphamide-Based Graft-Versus-Host Disease Prophylaxis Attenuates Disparity in Outcomes Between Use of Matched or Mismatched Unrelated Donors. J Clin Oncol. 2024;42:3277–86. 39018507 10.1200/JCO.24.00184 PMC11421565

[29] Penack O, Marchetti M, Aljurf M, Arat M, Bonifazi F, Duarte RF, et al. Prophylaxis and management of graft-versus-host disease after stem-cell transplantation for haematological malignancies: updated consensus recommendations of the European Society for Blood and Marrow Transplantation. Lancet Haematol. 2024;11:e147–e59. 38184001 10.1016/S2352-3026(23)00342-3

[30] Kekre N, Mak KS, Stopsack KH, Binder M, Ishii K, Brånvall E, et al. Impact of HLA-Mismatch in Unrelated Donor Hematopoietic Stem Cell Transplantation: A Meta-Analysis. Am J Hematol. 2016;91:551–5. 26927727 10.1002/ajh.24342

[31] Lee SJ, Klein J, Haagenson M, Baxter-Lowe LA, Confer DL, Eapen M, et al. High-resolution donor-recipient HLA matching contributes to the success of unrelated donor marrow transplantation. Blood. 2007;110:4576–83. 17785583 10.1182/blood-2007-06-097386

[32] Fürst D, Müller C, Vucinic V, Bunjes D, Herr W, Gramatzki M, et al. High-resolution HLA matching in hematopoietic stem cell transplantation: a retrospective collaborative analysis. Blood. 2013;122:3220–9. 24046013 10.1182/blood-2013-02-482547

[33] Ayuk F, Beelen DW, Bornhäuser M, Stelljes M, Zabelina T, Finke J, et al. Relative Impact of HLA Matching and Non-HLA Donor Characteristics on Outcomes of Allogeneic Stem Cell Transplantation for Acute Myeloid Leukemia and Myelodysplastic Syndrome. Biol Blood Marrow Transpl. 2018;24:2558–67. 10.1016/j.bbmt.2018.06.026 29966760

[34] Tie R, Zhang T, Yang B, Fu H, Han B, Yu J, et al. Clinical implications of HLA locus mismatching in unrelated donor hematopoietic cell transplantation: a meta-analysis. Oncotarget. 2017;8:27645–60. 28206973 10.18632/oncotarget.15291 PMC5432365

[35] Fleischhauer K, Tran TH, Meisel R, Mytilineos J, Dreger P, Kröger N. Donor Selection for Allogeneic Hematopoietic Cell Transplantation. Dtsch Arztebl Int. 2023;120:261–8. 36949660 10.3238/arztebl.m2023.0031 PMC10366961

[36] Bacigalupo A, Ballen K, Rizzo D, Giralt S, Lazarus H, Ho V, et al. Defining the intensity of conditioning regimens: working definitions. Biol Blood Marrow Transpl. 2009;15:1628–33. 10.1016/j.bbmt.2009.07.004 PMC2861656 19896087

[37] Gyurkocza B, Sandmaier BM. Conditioning regimens for hematopoietic cell transplantation: one size does not fit all. Blood. 2014;124:344–53. 24914142 10.1182/blood-2014-02-514778 PMC4102707

[38] Przepiorka D, Weisdorf D, Martin P, Klingemann HG, Beatty P, Hows J, et al. 1994 Consensus Conference on Acute GVHD Grading. Bone Marrow Transpl. 1995;15:825–8. 7581076

[39] Filipovich AH, Weisdorf D, Pavletic S, Socie G, Wingard JR, Lee SJ, et al. National Institutes of Health consensus development project on criteria for clinical trials in chronic graft-versus-host disease: I. Diagnosis and staging working group report. Biol Blood Marrow Transpl. 2005;11:945–56. 10.1016/j.bbmt.2005.09.004 16338616

[40] Jagasia MH, Greinix HT, Arora M, Williams KM, Wolff D, Cowen EW, et al. National Institutes of Health Consensus Development Project on Criteria for Clinical Trials in Chronic Graft-versus-Host Disease: I. The 2014 Diagnosis and Staging Working Group report. Biol Blood Marrow Transpl. 2015;21:389–401.e1. 10.1016/j.bbmt.2014.12.001 PMC4329079 25529383

[41] Sorror ML, Maris MB, Storb R, Baron F, Sandmaier BM, Maloney DG, et al. Hematopoietic cell transplantation (HCT)-specific comorbidity index: a new tool for risk assessment before allogeneic HCT. Blood. 2005;106:2912–9. 15994282 10.1182/blood-2005-05-2004 PMC1895304

[42] Gratwohl A. The EBMT risk score. Bone Marrow Transpl. 2012;47:749–56. 10.1038/bmt.2011.110 21643021

[43] Gu Z, Wang L, Yuan L, Huang W, Li M, Guan L, et al. Similar outcomes after haploidentical transplantation with post-transplant cyclophosphamide versus HLA-matched transplantation: a meta-analysis of case-control studies. Oncotarget. 2017;8:63574–86. 28969012 10.18632/oncotarget.18862 PMC5609944

[44] McCurdy SR, Kasamon YL, Kanakry CG, Bolaños-Meade J, Tsai HL, Showel MM, et al. Comparable composite endpoints after HLA-matched and HLA-haploidentical transplantation with post-transplantation cyclophosphamide. Haematologica. 2017;102:391–400. 27846611 10.3324/haematol.2016.144139 PMC5286947

[45] Rashidi A, Hamadani M, Zhang MJ, Wang HL, Abdel-Azim H, Aljurf M, et al. Outcomes of haploidentical vs matched sibling transplantation for acute myeloid leukemia in first complete remission. Blood Adv. 2019;3:1826–36. 31201170 10.1182/bloodadvances.2019000050 PMC6595262

[46] Arrieta-Bolaños E, Bonneville EF, Crivello P, Robin M, Gedde-Dahl T, Salmenniemi U, et al. Human Leukocyte Antigen Mismatching and Survival in Contemporary Hematopoietic Cell Transplantation for Hematologic Malignancies. J Clin Oncol. 2024;42:3287–99. 39167735 10.1200/JCO.24.00582

[47] Baker M, Wang H, Rowley SD, Cai L, Pecora AL, Skarbnik A, et al. Comparative Outcomes after Haploidentical or Unrelated Donor Bone Marrow or Blood Stem Cell Transplantation in Adult Patients with Hematological Malignancies. Biol Blood Marrow Transpl. 2016;22:2047–55. 10.1016/j.bbmt.2016.08.003 27522040

[48] Nagler A, Labopin M, Arat M, Reményi P, Koc Y, Blaise D, et al. Posttransplant cyclophosphamide-based anti-graft-vs-host disease prophylaxis in patients with acute lymphoblastic leukemia treated in complete remission with allogeneic hematopoietic cell transplantation from human leukocyte antigen-mismatched unrelated donors versus haploidentical donors: A study on behalf of the ALWP of the EBMT. Cancer. 2022;128:3959–68. 36110063 10.1002/cncr.34452

[49] Baron F, Labopin M, Tischer J, Ciceri F, Raiola AM, Blaise D, et al. Comparison of HLA-mismatched unrelated donor transplantation with post-transplant cyclophosphamide versus HLA-haploidentical transplantation in patients with active acute myeloid leukemia. Bone Marrow Transpl. 2022;57:1657–63. 10.1038/s41409-022-01781-9 35978005

[50] Crivello P, Arrieta-Bolaños E, He M, Wang T, Fingerson S, Gadalla SM, et al. Impact of the HLA Immunopeptidome on Survival of Leukemia Patients After Unrelated Donor Transplantation. J Clin Oncol. 2023;41:2416–27. 36669145 10.1200/JCO.22.01229 PMC10150892

[51] Fuchs EJ, McCurdy SR, Solomon SR, Wang T, Herr MM, Modi D, et al. HLA informs risk predictions after haploidentical stem cell transplantation with posttransplantation cyclophosphamide. Blood. 2022;139:1452–68. 34724567 10.1182/blood.2021013443 PMC8914182

[52] Stölzel F ML, Bug G, Kaufmann M, Bethge W, Stelljes M, et al., editor The randomized controlled HAMLET trial shows no survival difference between haploidentical related and single HLA-loci mismatched unrelated donor transplantation. 32nd European Hematology Association (EHA) Congress; 2025 June 12–15, 2025; Milan, Italy: HemaSphere.

