# Supplementary material for: Haploidentical transplantation with post-transplant cyclophosphamide is not inferior to 9/10-MUD transplantation with ATG in patients with myeloid malignancies
Source: Bone Marrow Transplant. 2026 Apr 8;61(6):694–701. doi: 10.1038/s41409-026-02827-y (PMC13241312; doi:10.1038/s41409-026-02827-y)
Supplement: Supplementary file 1 — Supplemental Material [file 41409_2026_2827_MOESM1_ESM.docx]

***Supplementary Materials***

**Haploidentical transplantation with post-transplant cyclophosphamide is not inferior to 9/10 MUD transplantation with ATG in high-risk myeloid malignancies.**

**A study on behalf of the German registry for hematopoietic stem cell transplantation and cell therapy (DRST).**

Andrea Gantner^1^, Svenja Labuhn^2^, Daniel Fürst^3,4^, Sophie Mannes^5^, Sarah Flossdorf^5,6^, Francis Ayuketang Ayuk^7^, Thomas Schroeder^8^, Matthias Stelljes^9^, Robert Zeiser^10^, Peter Dreger^11^, Matthias Eder^12^, Igor Wolfgang Blau^13^, Johannes Schetelig^,14^, Arne Brecht^15^, Andreas Burchert^16^, Matthias Edinger^17^, Verena Wais^1^, Hubert Schrezenmeier^3,4^, Hartmut Döhner^1^, Sandra Schmeller^2^, Katharina Fleischhauer^5,18^, Nicolaus Kröger^5,7^ Elisa Sala^1^; [German Registry for Hematopoietic Stem Cell Transplantation and cell Therapy, DRST](https://pubmed.ncbi.nlm.nih.gov/?sort=date&term=German+Registry+for+Stem+Cell+Transplantation%2C+DRST%5BCorporate+Author%5D).

This Supplementary Material provides additional methodological details, figures, and tables supporting the results presented in the main manuscript.

**Supplementary Methods**

The propensity score matching (PSM) was performed as a sensitivity analysis to assess the robustness of the results obtained in the original multivariable Cox proportional hazards model.

Matching was conducted using the R package MatchIt (version 4.7.2), applying 1:1 nearest-neighbor matching without replacement. Balance after matching was assessed using standardized mean differences and visualized using a Love plot (**Figure S1**), which also displays the variables included in the propensity score model.

In the propensity score–matched cohort, post-transplant major outcomes (OS, PFS, and GRFS) were analyzed using a Cox proportional hazards model accounting for the matched-pair design to investigate the influence of donor type and GVHD prophylaxis (9/10 MUD + ATG versus haplo + PT-Cy) on these endpoints (**Table S1**).

**Supplementary Figures**

**Figure S1.** Love plot showing standardized mean differences for baseline covariates before (open circles) and after (filled circles) propensity score matching. Covariates included in the propensity score model are shown. Matching improved balance between groups for most covariates.

**
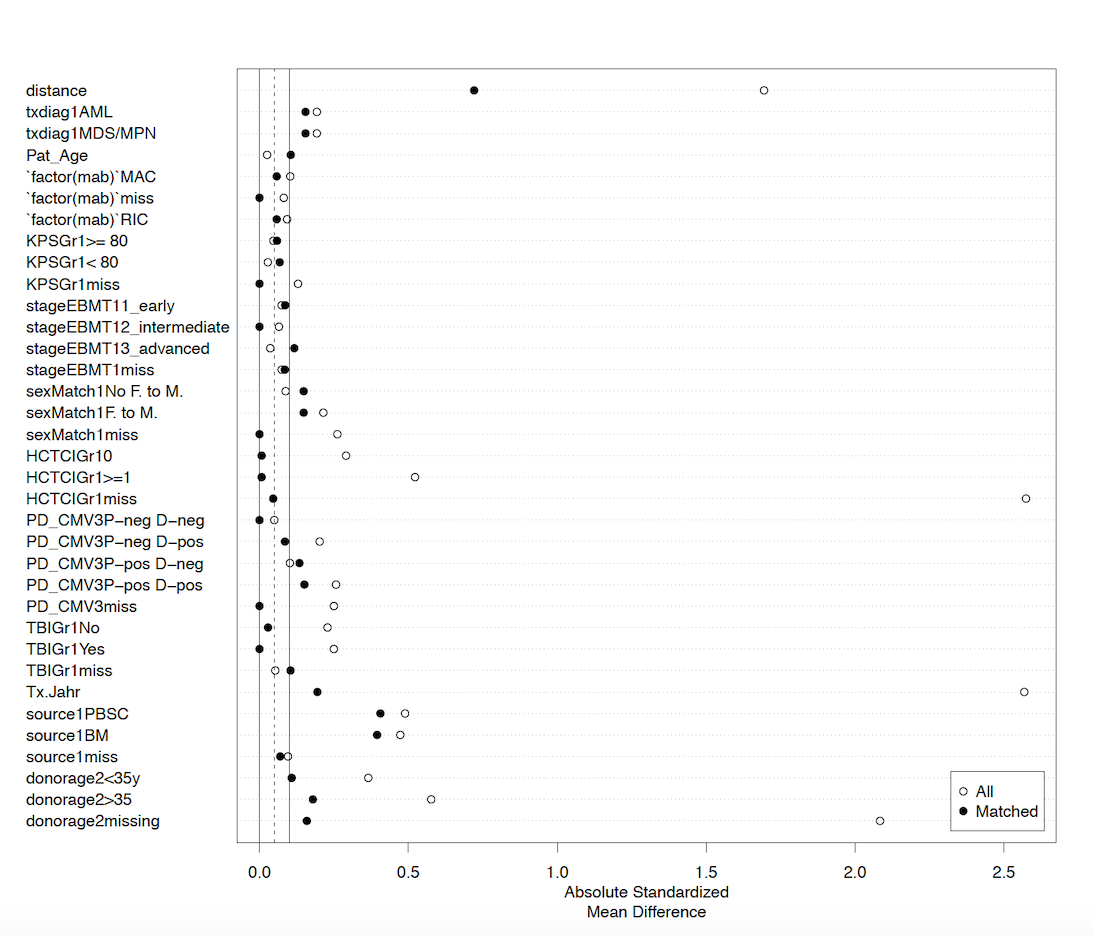
**

**Abbreviations**: AML= acute myeloid leukemia; MDS/MPN= myelodysplastic syndrome/myeloproliferative neoplasia; MAC=Myeloablative conditioning; RIC=Reduced intensity conditioning; KPS= Karnofsky Performance Status; HCT-CI= hematopoietic cell transplantation – comorbidity index; CMV= cytomegalovirus; TBI=Total body irradiation; PBSC=Peripheral blood stem cells; BM=Bone Marrow.

**Supplementary Tables**

**Table S1**. Matched pair analysis of factors influencing overall survival (OS), progression free survival (PFS) and GvHD-free-relapse-free survival (GRFS) after allogenic stem cell transplantation (allo-SCT).

|  | OS | | | PFS | | | GRFS | | |
| --- | --- | --- | --- | --- | --- | --- | --- | --- | --- |
|  | **HR** | **95% CI** | **P value** | **HR** | **95% CI** | **P value** | **HR** | **95% CI** | **P value** |
| Donor type |  |  |  |  |  |  |  |  |  |
| 9/10-MUD +ATG | - | - |  | - | - |  | - | - |  |
| Haplo + PT-Cy | 1.16 | 0.89-1.51 | 0.275 | 1.19 | 0.92-1.52 | 0.179 | 1.09 | 0.88-1.35 | 0.424 |
| Patient age at allo-SCT | 1.03 | 1.02-1.04 | **<0.001** | 1.02 | 1.01-1.03 | **0.001** | 1.01 | 1-1.02 | 0.123 |
| Disease status at allo-SCT* |  |  |  |  |  |  |  |  |  |
| early | - | - |  | - | - |  | - | - |  |
| Intermediate | 1.11 | 0.79-1.54 | 0.574 | 1.15 | 0.85-1.57 | 0.388 | 0.96 | 0.73-1.26 | 0.783 |
| advanced | 1.74 | 1.3-2.34 | **<0.001** | 1.75 | 1.32-2.31 | **<0.001** | 1.63 | 1.27 -2.1 | **<0.001** |
| KPS <80 | 2.01 | 1.33-3.04 | **0.001** | 1.9 | 1.28-2.81 | **0.001** | 1.29 | 0.89-1.87 | 0.172 |
| Stem cell source |  |  |  |  |  |  |  |  |  |
| PBSC | - | - |  | - | - |  | - | - |  |
| BM | 0.74 | 0.5-1.1 | 0.133 | 0.75 | 0.52-1.09 | 0.127 | 0.78 | 0.57-1.07 | 0.125 |
| Sex mismatch (donor/patient) |  |  |  |  |  |  |  |  |  |
| others | - | - |  | - | - |  | - | - |  |
| Female to male | 1.52 | 1.12-2.08 | **0.008** | 1.23 | 0.91-1.66 | 0.184 | 1.32 | 1.01-1.72 | **0.043** |
| Donor age |  |  |  |  |  |  |  |  |  |
| >35 years | **1.36** | **1.04-1.79** | **0.026** | 1.43 | 1.11-1.85 | **0.006** | 1.12 | 0.9-1.4 | 0.297 |
| Conditioning regimen |  |  |  |  |  |  |  |  |  |
| MAC | - | - |  | - | - |  | - | - |  |
| RIC | 1.3 | 1-1.7 | 0.053 | 1.24 | 0.97-1.6 | 0.091 | 1.2 | 0.96-1.5 | 0.104 |
| CMV status (patient/donor) |  |  |  |  |  |  |  |  |  |
| neg/neg | - | - |  | - | - |  | - | - |  |
| neg/pos | 1.44 | 0.84-2.49 | 0.188 | 1.46 | 0.87-2.43 | 0.148 | 1.13 | 0.73-1.74 | 0.585 |
| pos/neg | 1.76 | 1.22-2.55 | **0.002** | 1.86 | 1.31-2.64 | **<0.001** | 1.3 | 0.96-1.75 | 0.09 |
| pos/pos | 1.58 | 1.12-2.23 | **0.01** | 1.62 | 1.17-2.24 | **0.003** | 1.23 | 0.94-1.61 | 0.137 |
| TBI |  |  |  |  |  |  |  |  |  |
| no | - | - |  | - | - |  | - | - |  |
| yes | 1.32 | 0.94-1.86 | 0.111 | 1.15 | 0.83-1.6 | 0.41 | 0.97 | 0.72-1.3 | 0.847 |
| Year of allo-SCT | 1.01 | 0.93-1.09 | 0.861 | 0.99 | 0.92-1.06 | 0.768 | 1 | 0.94-1.06 | 0.97 |

**Abbreviations**: MUD= matched unrelated donor; ATG= anti-thymocyte globulin; Haplo= haploidentical donor; PT-CY=Post transplant cyclophosphamide; allo-SCT= allogeneic stem cell transplantation; KPS= Karnofsky Performance Status; PBSC=Peripheral blood stem cells; BM=Bone Marrow; MAC=Myeloablative conditioning; RIC=Reduced intensity conditioning; TBI=Total body irradiation.

^*^According to the classification present in the EBMT-risk score.

**Table S2**. Multivariable analysis of factors influencing overall survival (OS), progression free survival (PFS) and GvHD-free-relapse-free survival (GRFS) after allogenic stem cell transplantation (allo-SCT). HLA Class I mismatch cohort.

|  | OS | | | PFS | | | GRFS | | |
| --- | --- | --- | --- | --- | --- | --- | --- | --- | --- |
|  | **HR** | **95% CI** | **P value** | **HR** | **95% CI** | **P value** | **HR** | **95% CI** | **P value** |
| Donor type |  |  |  |  |  |  |  |  |  |
| 9/10-MUD + ATG | - | - |  | - | - |  | - | - |  |
| Haplo + PT-Cy | 1.18 | 0.92-1.52 | 0.201 | 1.2 | 0.94-1.53 | 0.138 | 1.13 | 0.91-1.39 | 0.267 |
| Patient age at allo-SCT | 1.02 | 1.01-1.03 | **<0.001** | 1.01 | 1.01-1.02 | **<0.001** | 1 | 1-1.01 | 0.418 |
| Disease status at allo-SCT* |  |  |  |  |  |  |  |  |  |
| early | - | - |  | - | - |  | - | - |  |
| Intermediate | 1.16 | 0.96-1.41 | 0.128 | 1.2 | 1-1.45 | 0.051 | 1.13 | 0.95-1.34 | 0.168 |
| advanced | 1.58 | 1.3-1.92 | **<0.001** | 1.6 | 1.33-1.93 | **<0.001** | 1.48 | 1.24-1.75 | **<0.001** |
| KPS <80 | 1.5 | 1.13-2 | **0.005** | 1.42 | 1.08-1.88 | **0.013** | 1.17 | 0.89-1.53 | 0.255 |
| Stem cell source |  |  |  |  |  |  |  |  |  |
| PBSC | - | - |  | - | - |  | - | - |  |
| BM | 0.87 | 0.65-1.17 | 0.354 | 0.91 | 0.69-1.2 | 0.502 | 1.09 | 0.85-1.39 | 0.498 |
| Sex mismatch (donor/patient) |  |  |  |  |  |  |  |  |  |
| others | - | - |  | - | - |  | - | - |  |
| Female to male | 1.29 | 1.04-1.6 | **0.023** | 1.21 | 0.98-1.49 | 0.081 | 1.19 | 0.98-1.45 | 0.083 |
| Donor age |  |  |  |  |  |  |  |  |  |
| >35 years | 1.28 | 1.03-1.59 | **0.029** | 1.24 | 1.01-1.52 | **0.043** | 1.1 | 0.92-1.33 | 0.305 |
| Conditioning regimen |  |  |  |  |  |  |  |  |  |
| MAC | - | - |  | - | - |  | - | - |  |
| RIC | 0.91 | 0.76-1.08 | 0.264 | 0.92 | 0.78-1.09 | 0.328 | 0.99 | 0.85-1.15 | 0.872 |
| CMV status (patient/donor) |  |  |  |  |  |  |  |  |  |
| neg /neg | - | - |  | - | - |  | - | - |  |
| neg/pos | 1.1 | 0.82-1.48 | 0.532 | 1.03 | 0.77-1.37 | 0.852 | 0.98 | 0.75-1.27 | 0.885 |
| pos/neg | 1.47 | 1.18-1.83 | **0.001** | 1.44 | 1.17-1.78 | **0.001** | 1.2 | 0.99-1.46 | 0.07 |
| pos/pos | 1.22 | 0.99-1.5 | 0.058 | 1.2 | 0.99-1.46 | 0.068 | 1.09 | 0.91-1.3 | 0.337 |
| TBI |  |  |  |  |  |  |  |  |  |
| none | - | - |  | - | - |  | - | - |  |
| Yes | 1.25 | 1.03-1.52 | **0.025** | 1.09 | 0.9-1.33 | 0.357 | 0.97 | 0.81-1.16 | 0.74 |
| Year of allo-SCT | 0.99 | 0.96-1.01 | 0.39 | 0.99 | 0.96-1.01 | 0.307 | 0.99 | 0.97-1.01 | 0.363 |

**Abbreviations**: MUD= matched unrelated donor; ATG= anti-thymocyte globulin; Haplo= haploidentical donor; PT-CY=Post transplant cyclophosphamide; allo-SCT= allogeneic stem cell transplantation; KPS= Karnofsky Performance Status; PBSC=Peripheral blood stem cells; BM=Bone Marrow; MAC=Myeloablative conditioning; RIC=Reduced intensity conditioning; TBI=Total body irradiation.

^*^According to the classification present in the EBMT-risk score.
