# Supplementary material for: Haploidentical transplantation with post-transplant cyclophosphamide is not inferior to 9/10-MUD transplantation with ATG in patients with myeloid malignancies
Source: Bone Marrow Transplant. 2026 Apr 8;61(6):694–701. doi: 10.1038/s41409-026-02827-y (PMC13241312; doi:10.1038/s41409-026-02827-y)
Supplement: Supplementary file 2 — Appendix [file 41409_2026_2827_MOESM2_ESM.docx]

**Appendix: DRST members contributing to this study**

Professor Nicolaus Kröger, Universitätsklinikum Hamburg-Eppendorf, 166 patients

Dr. Thomas Schroeder, Universitätsklinikum Essen, 157 patients

Professor Matthias Stelljes, Universitätsklinikum Münster, 79 patients

Professor Robert Zeiser, Universitätsklinikum Freiburg, 78 patients

Professor Peter Dreger, Universitätsklinikum Heidelberg, 67 patients

Professor Matthias Eder, Med. Hochschule Hannover, 65 patients

Professor Igor Wolfgang Blau, Charité - Universitätsmedizin Berlin, 60 patients

Professor Johannes Schetelig, Universitätsklinikum Carl Gustav Carus a. d. TU Dresden, 59 patients

Dr. Arne Brecht, DKD HELIOS Klinik Wiesbaden Zentrum f. Blutstammzell- u. Knochenmarktransplantation, 48 patients

Professor Matthias Edinger, Universitätsklinikum Regensburg, 45 patients

Professor Andreas Burchert, Universitätsklinik Marburg, 45 patients

Dr. Gesine Bug, Universitätsklinikum Frankfurt (Main), 44 patients

Dr. Daniel Teschner, Universitätsklinikum Würzburg, 44 patients

Professor Wolfgang Bethge, Universitätsklinikum Tübingen, 41 patients

Professor Inken Hilgendorf, Universitätsklinikum Jena, 38 patients

Dr. Julia Winkler, Universitätsklinikum Erlangen, 35 patients

Dr. Martin Kaufmann, Robert-Bosch-Krankenhaus Stuttgart, 28 patients

Dr. Jörg Thomas Bittenbring, Universitätsklinikum des Saarlandes, 26 patients

Professor Friedrich Stölzel, Universitätsklinikum Kiel, 26 patients

Dr. Eva Wagner-Drouet, Universitätsmedizin Mainz, 26 patients

Professor Uwe Platzbecker, Universitätsklinikum Leipzig, 25 patients

Dr. Mareike Verbeek, Klinikum rechts der Isar der TU München, 18 patients

Professor Christof Scheid, Universitätsklinikum Köln, 16 patients

Professor Gerald Wulf, Universitätsklinik Göttingen, 16 patients

Dr. Johanna Tischer, Klinikum der Universität München -Großhadern, 15 patients

Dr. Frederike Wortmann, Universitätsklinikum Schleswig-Holstein / Campus Lübeck, 13 patients

Dr. Elisa Sala, Universitätsklinikum Ulm, 13 patients

Professor William Krüger, Universitätsklinikum Greifswald, 12 patients

Professor Michael Kiehl, Klinikum Frankfurt (Oder), 12 patients

Professor Edgar Jost, Universitätsklinik RWTH Aachen, 11 patients

Dr. Mareike Dürholt, KEM - Kliniken Essen-Mitte, Evang. Krankenhaus Essen-Werden, 10 patients

Dr. Tobias Holderried, Universitätsklinikum Bonn, 9 patients

Professor Ahmet Elmaagacli, Asklepios Klinik St. Georg Hamburg, 9 patients

Professor Lutz P. Müller, Universitätsklinikum Halle (Saale), 8 patients

Dr. Stefan Klein, Universitätsmedizin Mannheim, 7 patients

Dr. Denise Walther, Universitätsklinikum Magdeburg, 7 patients

Professor Guido Kobbe, Universitätsklinikum Düsseldorf, 7 patients

Professor Angela Krackhardt, Malteser Krankenhaus Flensburg, 6 patients

Professor Christoph Schmid, Universitätsklinikum Augsburg, 5 patients

Professor Jochen Casper, Klinikum Oldenburg AöR, 4 patients

Professor Axel Fauser, Klinik für KMT und Hämatologie/Onkologie GmbH, 3 patients

Dr. Judith Niederland, Helios Klinikum Berlin-Buch, 3 patients

Professor Roland Schroers, Ruhr Universität Bochum, 2 patients

Dr. Tobias Bartscht, Helios Klinikum Schwerin, 2 patients

Professor Mark Ringhoffer, Städt. Klinikum Karlsruhe gGmbH, 1 patient

Dr. Stefan Kaun, Klinikum Bremen-Mitte, 1 patient

Professor Matthias Wölfl, Universitätsklinikum Würzburg Kinderklinik u. Poliklinik, 1 patients
